# The impact of caring for family members with mental illnesses on the caregiver: a scoping review

**DOI:** 10.1093/heapro/daac049

**Published:** 2022-04-26

**Authors:** Rita Phillips, Mark Durkin, Hilary Engward, Graham Cable, Maria Iancu

**Affiliations:** 1 Robert Gordon University, Garthdee House, Garthdee Road, Aberdeen AB10 7QB, UK; 2 Leeds Trinity University, Leeds, UK; 3 Anglia Ruskin University, Cambridge Campus, East Rd, Cambridge CB1 1PT, UK

**Keywords:** family caregiving, scoping review, mental health, family carers

## Abstract

A large number of multidisciplinary, qualitative and quantitative research suggests that providing care for family members with mental health illnesses can have both positive and negative effects on the carers’ wellbeing. However, to date a comprehensive overview and synthesis of literature that compares and contrasts positive and negative effects of family-caregiving on the carer is missing. To address this gap, this scoping review examines the effects of family-caregiving on carers’ wellbeing. A Boolean search generated a total of 92 relevant articles that were included in the analysis. The results suggest that, to understand the effects of family-caregiving on the carer’s mental and physical wellbeing, it is necessary to take a combination of situational and sociodemographic characteristics into consideration. Elderly, female, spousal-carers and primary-carers may be a group that is at risk of suffering from a lack of positive mental and physical wellbeing as a result of caring. However, the negative effects of caregiving can be balanced by extraversion, social support and religious or spiritual beliefs. Therefore, future interventions that aim to promote family caregivers’ wellbeing may need to take personality, particular circumstances as well as cultural and personal beliefs into consideration.

## INTRODUCTION

According to recent studies, an increasing number of people experience mental health disorders (Fernando, 2014; Bruffaerts *et al.*, 2015; Polanczyk *et al.*, 2015; Hanna *et al.*, 2018). Due to a global deinstitutionalization of the treatment of mental illnesses, only a low proportion of those suffering from mental illnesses are admitted to hospitals (WHO, 2017). Of those who are hospitalized, ∼30–50% experience a relapse of symptoms within the first 6 months and 50–70% in the first 5 years after being discharged (Chang and Chou, 2015; Ali *et al.*, 2017; Sadock *et al.*, 2017). Comorbidity with other mental illness, non-adherence to medication, shorter duration on treatment and experiencing stressful life events as well as high disability score, and a single admission history are significant predictors of mental health relapse (Agenagnew and Kassaw, 2020; Moges *et al.*, 2021). Patients with psychotic disorders who also experience common mental health disorders such as depression and anxiety are more likely to experience a relapse (Ali *et al.*, 2017; Moges *et al.*, 2021). Some novel approaches such as receiving low-intensity personalized advice via text-messages post-treatment (Malins *et al.*, 2020) and specific forms of therapy such as Acceptance and Commitment Therapy (Østergaard, 2020), mind–body relaxation and therapies that allow the patient to develop healthy coping skills (Melemis, 2015) can be particularly beneficial in relapse prevention. However, as family and spousal carers live with patients and are usually the first to recognize behavioural changes, they play an increasingly important role in supporting and rehabilitating those who suffer from mental health illnesses.

The challenges that family members who care for those with mental health illnesses face are well documented in the literature. Family caregivers are at an increased risk of suffering physically, psychologically and socially while providing care for family members with mental health conditions (Sharma *et al.,* 2016; Akbari *et al.*, 2018; Norris *et al.*, 2018; Özgönül and Bademli, 2022). Specifically, studies have shown that caring for family members with mental health problems can lead to social isolation, financial difficulties, occupational restrictions and negative emotions such as anger, aggression, frustration, low self-esteem, constant worry and feelings of helplessness (van der Sanden *et al.*, 2013, 2015; Yin *et al.*, 2014; Lamont and Dickens, 2021). In addition, there is an increased risk of reduced life-expectancy, lower wellbeing and mastery of life skills, and less time spent doing leisure activities for family caregivers (Akbari *et al.*, 2018; Hsiao *et al.*, 2020; McKee, 2020; Dadalto and Cavalcante, 2021).

Yet, research also shows that caring for a family member may also affect the caregivers in positive ways (Roth *et al.*, 2015). For example, literature suggests that some family carers can become more resilient over time (Crellin *et al.*, 2014; Donnellan *et al.*, 2015; Ong *et al.*, 2018; Post *et al.*, 2021). While there are different definitions for caregiver resilience in the literature, they all share similar descriptions for the characteristics for overcoming adversity. This is explained as not only being about surviving the burden associated with caring for a mentally ill family member, but also growing into a stronger, more adaptable and healthier person (Amagai *et al.*, 2016; Pione *et al.*, 2021). In addition, family caregiving can improve the relationship between the caregiver and the person they care for, while also providing a sense of inner strength and satisfaction (Kate *et al.*, 2013; Roth *et al.*, 2015; Grover *et al.*, 2017; Shiraishi and Reilly, 2019).

While previous research examined possible challenges and advantages of caring for family members with mental health problems, a comprehensive overview and synthesis of the literature appears to be missing. This is problematic, as there is a need to understand the unique challenges that family carers face and determine possible sources of help and support that would allow for implementation of informed strategies to meet the carers’ needs. The present study addresses this gap by reviewing empirical studies and scientific evidence to capture both the challenges and positive aspects of caregiving in the family context.

## METHODS

A scoping review was used to synthesize the relevant literature on family caregiving (Arksey and O’Malley, 2005; Levac *et al.*, 2010). Scoping reviews are a ‘useful way of mapping fields of study where it is difficult to visualize the range of material that might be available’ [(Arksey and O’Malley, 2005), p. 21]. They allow for a synthesis of the literature across different academic disciplines. This technique was relevant to the present study as empirical inquiry surrounding positive and negative effects of family caregiving are the subject of interest in various disciplines. For example, clinical studies might examine the physical and mental health effects of family caregiving, and psychological studies observe the severity of caregiver burden or changes in identity formation, whereas sociological studies tend to focus on the effects of family caregiving on social structures such as families or friendship networks. A scoping review utilizes a rigorous methodological framework consisting of five steps, which includes contributions from several disciplines. It maps the terrain of existing research and highlights gaps in knowledge (Arksey and O’Malley, 2005; Levac *et al.*, 2010). As a method for reviewing literature, scoping studies have distinct characteristics (Arksey and O’Malley, 2005). Unlike systematic reviews, they address broader topics and topic areas in which many different study designs might be applicable (Arksey and O’Malley, 2005). Therefore, this approach was suitable to identify relevant themes in family caregiving in relation to caregiver health and wellbeing. The present study utilized Arksey and O’Malley’s (Arksey and O’Malley, 2005) five-step protocol of scoping reviews because this is one of the most frequently utilized and published frameworks in scoping review literature (i.e. Abraham *et al.*, 2010; Halas *et al.*, 2015; O’Flaherty and Phillips, 2015; O’Brien *et al.*, 2016; Walsh *et al.*, 2019). The five steps include: (i) identifying the research question, (ii) identifying relevant studies, (iii) study selection, (iv) charting the data and (v) collating, summarizing and reporting the results. Each step is outlined in more detail below.

### Step 1: Identifying the research question

The first methodological step when conducting a scoping review is to determine the focus of the research question (Arksey and O’Malley, 2005; Levac *et al.*, 2010). A research team from Robert Gordon University (Aberdeen), Anglia Ruskin University and the Forces in Mind Research Centre (Chelmsford) considered the appropriate balance of breadth and depth for the research topics (Arksey and O’Malley, 2005; Levac *et al.*, 2010) during the literature review. The following questions were determined in December 2020 and guided the scoping review:

“How does family caregiving affect the caregiver physically, mentally and socially?” and

“How can family carers be best supported?”.

### Step 2: Identifying relevant studies

Guidelines concerning the identification of the literature in scoping reviews, include the development of search terms, identification of databases, and the establishment of time frames, were followed (Levac *et al.*, 2010). Therefore, the present review drew on a broad interpretation of search strings related to family caring and wellbeing. The social and health sciences databases APA Psych Articles, Web of Science, Science Direct, Scopus, Springer Link, SAGE Discipline Hub: Psychology and Counselling, Medline, SocINDEX, CINAHL and PTSDpubs were accessed. Additional studies were identified by manual searching the reference lists of the reviewed articles. The selection of articles was limited to peer-reviewed, English-language empirical studies published between January 2001 and March 2021 to ensure the recency of empirical evidence. Databases were searched by combining the search strings on family caregiving and the physical, mental and social consequences of caring. The key words were ‘Mental Health’ with the search strings ‘Mental health problem*’, ‘Mental health issue*’, ‘Ill-mental health’, ‘Mental health disorder*’, ‘Mental Distress’ and ‘Family Care’ with the search strings ‘Spousal carer*/ing’, ‘Informal care*/caring’, ‘Family care*/caring’, ‘Unpaid care*/caring’, ‘Care for family members with –’, ‘Support of family members with’ (cf. Table 1).

**Table 1:** Key words and search strings

Key words	Search strings
Mental health	Mental health problem* Mental health issue* Ill-mental health Mental health disorder* Mental Distress
Family care	Spousal carer*/ing Informal care*/caring Family care*/caring Unpaid care*/caring Care for family members with— Support of family members with—

### Step 3: Study selection

**Fig. 1:**
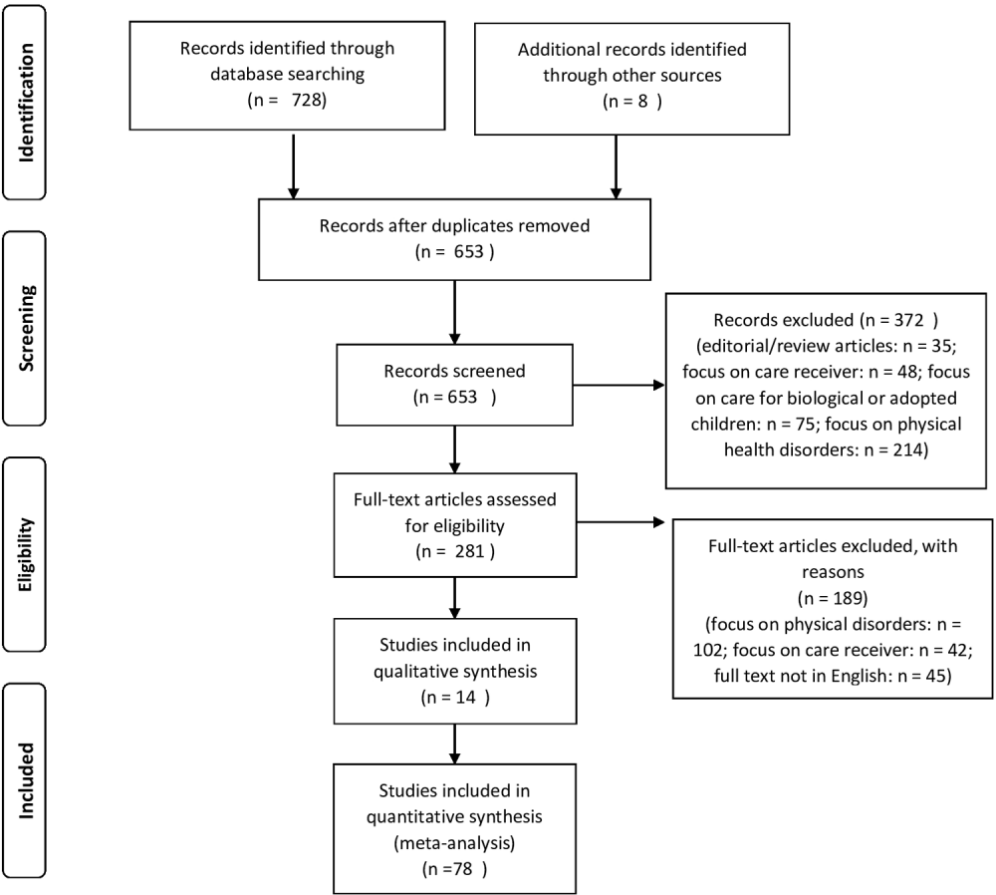
PRISMA flow diagram (cf. Moher *et al.*, 2009).

Decisions surrounding the inclusion and exclusion of studies in a scoping review rely upon an iterative and collaborative process involving all members of the researcher team (Levac *et al.*, 2010). To address this we used the *Preferred Reporting Items for Systematic Reviews* flow diagram (cf. Figure 1; Moher *et al.*, 2009). Databases were searched with a combination of search strings and Boolean operators to ensure the inclusion of at least one search string from the terms listed in Table 1. Both qualitative and quantitative studies were included. The exclusion criteria were the publication being an editorial or review article, studies with a primary focus on the care receiver and not the family carer, the patient being a child or adolescent (under 18 years of age) and studies about caring for physical health disorders. Editorial and review articles were examined to ensure the complete retrieval of relevant primary research noted therein.

### Step 4: Charting the data

In scoping reviews, charting the data involves reviewing, documenting and sorting the information that was retrieved in accordance with the key issues and themes (Arksey and O’Malley, 2005). An Excel spreadsheet was used to extract, document and organize the relevant information from each reviewed article (Levac *et al.*, 2010). The spreadsheet included separate columns for article title, authors, database from which the article was drawn, the population and social-cultural background, mental illness of the patient whom family carers supported, methodological framework, instruments used in the study, method of analysis, main findings, implications and any additional information (e.g. screenshots of tables and figures).

### Step 5: Collating, summarizing and reporting the results

The articles were read independently by members of the research team and topics relevant to the purpose of the review were identified. The research team compared, summarized and collated the identified preliminary themes. The data were then re-examined and reorganized to reflect deeper and more nuanced perspectives of the studies’ findings. Based on common meanings and central issues, the findings for each study were organized and integrated into categories and themes to highlight prevalent issues and gaps in knowledge. The research team commenced with collating, summarizing and reporting the results in April 2021 and concluded this step in August 2021.

## RESULTS

Boolean searches including the string search terms generated a total output of 736 articles. From these 736 articles, 83 were excluded as they were duplicates. The research team examined the remaining 653 articles for inclusion/exclusion independently. Concordance in interrater reliability was high with the researcher team agreeing on inclusion and exclusion of 646 of the 653 articles (98.93%). Arguments for inclusion or exclusion of the remaining seven articles (1.07%) were discussed and consensus on inclusion/exclusion was reached before commencing with further analyses. The initial screening involved reading the abstracts and skimming the tests, which identified 281 articles that could potentially qualify for inclusion in the review. These 281 articles were read in detail. From the 231 articles, a total of 189 were excluded from further review, based on the exclusion criteria (cf. Table 1). Therefore, a total of 92 studies were included in the present review, of which 14 were qualitative and 78 were quantitative studies. Most studies focussed on American or British participants. Other countries represented were Australia, Canada, Chile, China, Colombia, Cyprus, Finland, Germany, Greece, India, Iran, Ireland, Italy, Japan, Malaysia, Malta, New Zealand, Norway, Portugal, Saudi Arabia, Spain and the Netherlands. Various mental health conditions were present in this review, the highest proportion being articles concerning family carers’ experiences with dementia (38 articles, 41.3%), followed by family carer’s experiences handling multiple and/or various mental health conditions (25 articles, 27.18%) and Alzheimer disease (13 articles, 14.13%). A smaller number of articles discussed family carers’ experiences with depression (3 articles, 3.26%), polytrauma in relation to traumatic brain injury (TBI; 3 articles, 3.26%), schizophrenia (2 articles, 2.17%), suicidal ideation (2 articles, 2.17%), post traumatic stress disorder (PTSD; 2 articles, 2.17%), psychosis (1 article, 1.09%), Parkinson disease psychosis (1 article, 1.09%), Postpartum psychiatric disorder (1 article, 1.09%) and bipolar disorder (1 article, 1.09%). An overview of the studies included in the review can be found in Table 2.

**Table 2:** Table of articles and papers included in the analysis

References	Participants	Disease	Method
Hahn, E. A., Boileau, N. R., Hanks, R. A., Sander, A. M., Miner, J. A. and Carlozzi, N. E. (2020). Health literacy, health outcomes, and the caregiver role in traumatic brain injury. *Rehabilitation Psychology*, **65**, 401–408. https://doi-org.ezproxy.rgu.ac.uk/10.1037/rep0000330	131 family caregivers	TBI	Quantitative
Brickell, T. A., Lippa, S. M., French, L. M., Gartner, R. L., Driscoll, A. E., Wright, M. M. and Lange, R. T. (2019). Service needs and health outcomes among caregivers of service members and veterans following TBI. *Rehabilitation Psychology*, **64**, 72–86. https://doi-org.ezproxy.rgu.ac.uk/10.1037/rep0000249	264 caregivers (95.8% female)—veterans/military	TBI	Quantitative
Griffin, J. M., Lee, M. K., Bangerter, L. R., Van Houtven, C. H., Friedemann-Sánchez, G., Phelan, S. M., Carlson, K. F. and Meis, L. A. (2017). Burden and mental health among caregivers of veterans with traumatic brain injury/polytrauma. *American Journal of Orthopsychiatry*, **87**, 139–148. https://doi-org.ezproxy.rgu.ac.uk/10.1037/ort0000207	564 caregivers—veterans	TBI and Polytrauma	Quantitative
Mausbach, B. T., Chattillion, E. A., Ho, J., Flynn, L. M., Tiznado, D., von Känel, R., Patterson, T. L. and Grant, I. (2014). Why does placement of persons with Alzheimer’s disease into long-term care improve caregivers’ well-being? Examination of psychological mediators. *Psychology and Aging*, **29**, 776–786. https://doi-org.ezproxy.rgu.ac.uk/10.1037/a0037626	126 spousal caregivers	Alzheimer	Longitudinal, Quantitative
Gonçalves-Pereira, M., Zarit, S. H., Cardoso, A. M., Alves da Silva, J., Papoila, A. L. and Mateos, R. (2020). A comparison of primary and secondary caregivers of persons with dementia. *Psychology and Aging*, **35**, 20–27. https://doi-org.ezproxy.rgu.ac.uk/10.1037/pag0000380.supp (Supplemental)	61 primary and 61 secondary family caregivers	Dementia	Quantitative
Meyer, O. L., Nguyen, K. H., Dao, T. N., Vu, P., Arean, P. and Hinton, L. (2015). The sociocultural context of caregiving experiences for Vietnamese dementia family caregivers. *Asian American Journal of Psychology*, **6**, 263–272. https://doi-org.ezproxy.rgu.ac.uk/10.1037/aap0000024.supp (Supplemental)	10 caregivers	Dementia	Qualitative
Otero, P., Torres, Á. J., Vázquez, F. L., Blanco, V., Ferraces, M. J. and Díaz, O. (2019). Does the disease of the person receiving care affect the emotional state of non-professional caregivers? *Frontiers in Psychology*, **10**, 1144.	491 caregivers	Multiple	Quantitative
Alzahrani, S. H., Fallata, E. O., Alabdulwahab, M. A., Alsafi, W. A. and Bashawri, J. (2017). Assessment of the burden on caregivers of patients with mental disorders in Jeddah, Saudi Arabia. *BMC Psychiatry*, **17**, 1–8.	377 caregivers	Mental health illnesses (various)	Quantitative
Chiang, C. Y., Lu, C. Y., Lin, Y. H., Lin, H. Y. and Sun, F. K. (2015). Caring stress, suicidal attitude and suicide care ability among family caregivers of suicidal individuals: a path analysis. *Journal of Psychiatric and Mental Health Nursing*, **22**, 792–800.	164 family caregivers	Suicidal ideation	Quantitative
Unwin, G. and Deb, S. (2011). Family caregiver uplift and burden: Associations with aggressive behavior in adults with intellectual disability. *Journal of Mental Health Research in Intellectual Disabilities*, **4**, 186–205.	44 family caregivers	Intellectual disability or aggressive behaviour	Quantitative
Choi, H. (2018). Giving or receiving spouse care and marital satisfaction among older Korean individuals. *Social Science & Medicine*.	3424 spousal caregivers—not specified	Care—not specified	Quantitative
Casarez, R. L., Barlow, E., Iyengar, S. M., Soares, J. C. and Meyer, T. D. (2019). Understanding the role of m-Health to improve well-being in spouses of patients with bipolar disorder. *Journal of Affective Disorders*, **250**, 391–396.	13 spousal caregivers	Bipolar disorder	Qualitative
Greenwood, N., Pound, C., Brearley, S. and Smith, R. (2019). A qualitative study of older informal carers’ experiences and perceptions of their caring role. *Maturitas*, **124**, 1–7.	44 family carers aged 70–87 years	Dementia or other mental illness	Qualitative
Alasmee, N. and Hasan (2020). Primary caregivers experience of anti-psychotic medication: a qualitative study. *Archives of Psychiatric Nursing*, **34**, 520–528.	21 family caregivers	Schizophrenia	Qualitative
Colvez, A., Joel, M. E., Ponton-Sanchez, A. and Royer, A. C. (2002). Health status and work burden of Alzheimer patients’ informal caregivers: comparisons of five different care programs in the European Union. *Health Policy*, **60**, 219–233.	322 spousal caregivers	Alzheimer	Quantitative
Anchan, V. and Janardhana, N. (2020). Transformation of attitude through brief psychoeducation program for the husbands of women with postpartum psychiatric disorders. *Asian Journal of Psychiatry*, **51**, 101841.	21 husbands of women with postpartum psychiatric disorder	Postpartum psychiatric disorders	Quantitative
Lai, F. H. Y., Yan, E. W. H., Tsui, W. S. and Yu, K. K. Y. (2020). A randomized control trial of activity scheduling for caring for older adults with dementia and its impact on their spouse care-givers. *Archives of Gerontology and Geriatrics*, **90**, 104167.	100 spouses	Dementia	Quantitative
Van Wijngaarden, B., Koeter, M., Knapp, M., Tansella, M., Thornicroft, G., Vázquez-Barquero, J. L. *et al.* (2009). Caring for people with depression or with schizophrenia: are the consequences different? *Psychiatry Research*, **169**(1), 62–69.	252 family/friends/partner caregivers for outpatients with depression and 151 family/friends/partner caregivers for outpatients with schizophrenia	Multiple	Quantitative
Dahlrup, B., Ekström, H., Nordell, E. and Elmståhl, S. (2015). Coping as a caregiver: a question of strain and its consequences on life satisfaction and health-related quality of life. *Archives of Gerontology and Geriatrics*, **61**, 261–270.	369 family carers	Multiple	Quantitative
Jones, S. M., Woodward, M. and Mioshi, E. (2019). Social support and high resilient coping in carers of people with dementia. *Geriatric Nursing*, **40**, 584–589.	108 informal carers; Spousal relationship was most Common (61%), as was carer co-residence with the person with dementia (78%).	Dementia	Quantitative
Highet, N., Thompson, M. and McNair, B. (2005). Identifying depression in a family member: the carers’ experience. *Journal of Affective Disorders*, **87**(1), 25–33.	37 carers partners (*n* = 15/37), parents (*n* = 19/37), and siblings (*n* = 3/37)	Clinical depression	Qualitative
Beeson, R. A. (2003). Loneliness and depression in spousal caregivers of those with Alzheimer’s disease versus non-caregiving spouses. *Archives of Psychiatric Nursing*, **17**, 135–143.	49 caregiving spouses and 52 non-caregiving control individuals	Alzheimer	Quantitative
Kabitsi, N. and Powers, D. V. (2002). Spousal motivations of care for demented older adults: a cross-cultural comparison of Greek and American female caregivers. *Journal of Aging Studies*, **16**, 383–399.	30 American women spousal carers, 44 Greek women spousal carers	Dementia	Quantitative
Mills, P. J., Ancoli-Israel, S., von Känel, R., Mausbach, B. T., Aschbacher, K., Patterson, T. L. *et al.* (2009). Effects of gender and dementia severity on Alzheimer’s disease caregivers’ sleep and biomarkers of coagulation and inflammation. *Brain, Behavior, and Immunity*, **23**, 605–610.	81 male and female spousal caregiver and 41 non-caregivers	Alzheimer	Quantitative
Teles, S., Ferreira, A. and Paúl, C. (2021). Access and retention of informal dementia caregivers in psychosocial interventions: a cross-sectional study. *Archives of Gerontology and Geriatrics*, **93**, 104289.	179 Portuguese caregivers—unspecified	Dementia	Quantitative
Morrison, V. and Williams, K. (2020). Gaining longitudinal accounts of carers’ experiences using IPA and photograph elicitation. *Frontiers in Psychology*, 11, 521382.	3 women caregivers	Dementia, stroke and ftd	Qualitative
Zwar, L., König, H. H. and Hajek, A. (2020). Psychosocial consequences of transitioning into informal caregiving in male and female caregivers: findings from a population-based panel study. *Social Science & Medicine*, 264, 113281.	13 333 informal carers—unspecified	Unspecified	Quantitative
Willis, P., Vickery, A. and Symonds, J. (2020). “You have got to get off your backside; otherwise, you’ll never get out”: older male carers’ experiences of loneliness and social isolation. *International Journal of Care and Caring*, **4**, 311–330.	25 men; British—five distinct sample groups, including: male carers; men who were single and living in urban or rural areas; men who identified as gay and were single or living alone; and men with hearing loss.	Various (including dementia, Alzheimer, physical disabilities)	Qualitative
Brites, R., Brandão, T., Moniz Pereira, F., Hipólito, J. and Nunes, O. (2020). Effects of supporting patients with dementia: a study with dyads. *Perspectives in Psychiatric Care*, **56**, 614–620.	36 informal caregivers and their 36 care receivers	Dementia	Quantitative
Mahomed, A. and Pretorius, C. (2020). Availability and utilization of support services for South African male caregivers of people with Alzheimer’s disease in low-income communities. *Dementia*, **20**, 633–652.	11 adult males who were familial caregivers	Alzheimer	Qualitative
Ruisoto, P., Contador, I., Fernandez-Calvo, B., Serra, L., Jenaro, C., Flores, N. *et al.* (2020). Mediating effect of social support on the relationship between resilience and burden in caregivers of people with dementia. *Archives of Gerontology and Geriatrics*, **86**, 103952.	283 primary and family caregivers in Spain	Dementia	Quantitative
Kamalzadeh, L., Salehi, M., Rashedi, V., Ahmadzad Asl, M., Malakouti, S. K., Seddigh, R. *et al.* (2020). Perceived burden of dementia care, clinical, psychological and demographic characteristics of patients and primary caregivers in Iran. *Applied Neuropsychology Adult*.	60 family caregiver and care-recipient pairs	Dementia	Quantitative
Tranvåg, O., Nåden, D. and Gallagher, A. (2019). Dignity work of older women caring for a husband with dementia at home. *Health Care for Women International*, **40**, 1047–1069.	6 Norwegian women spousal carers	Dementia	Qualitative
Hvidsten, L., Engedal, K., Selbæk, G., Wyller, T. B., Benth, J.Š., Kersten, H.	88 family carers—unspecified	Dementia	Quantitative
Avdikou, K., Stefanatos, C., Tsatali, M., Gouva, M. and Tsolaki, M. (2019). The role of gender in shame, hostility, and aggression experienced by caregivers for patients with dementia. *American Journal of Alzheimer’s Disease & Other Dementias*, **34**, 231–235.	55 family caregivers	Dementia	Quantitative
Care recipients’ low level of cognitive function was associated with greater perceived burden. Higher quality of support was associated with lower perceived burden among female and male spouse caregivers, daughter caregivers and mother	20 207 respondents and 43 262 observations of 50+ carers	Unspecified	Quantitative
Mantri, S., Edison, B., Alzyoud, L., Marras, C., Chahine, L. M.	145 partner caregivers	Parkinson disease psychosis (PDP)	Mixed-methods cross-sectional study
Juntunen, K., Salminen, A. L., Törmäkangas, T., Tillman, P., Leinonen, K. and Nikander, R. (2018). Perceived burden among spouse, adult child, and parent caregivers. *Journal of Advanced Nursing*, **74**, 2340–2350.	4000 family caregivers in Finland	Unspecified	Quantitative
Dam, A. E., Boots, L. M., Van Boxtel, M. P., Verhey, F. R. and De Vugt, M. E. (2018). A mismatch between supply and demand of social support in dementia care: a qualitative study on the perspectives of spousal caregivers and their social network members. *International Psychogeriatrics*, **30**, 881–892.	10 spousal caregivers of people with dementia and 17 network members	Dementia	Qualitative
Beks, T. A. and Cairns, S. L. (2018). Contexts precipitating help-seeking among partners of veterans with posttraumatic stress disorder: a qualitative exploration. *Traumatology*, **24**, 313.	16 Canadian female partners (English-speaking) of male veterans with PTSD	Veterans/PTSD	Qualitative
Rahmani, F., Ebrahimi, H., Seyedfatemi, N., Namdar Areshtanab, H., Ranjbar, F. and Whitehead, B. (2018). Trapped like a butterfly in a spider’s web: experiences of female spousal caregivers in the care of husbands with severe mental illness. *Journal of Clinical Nursing*, **27**, 1507–1518.	14 female spousal caregivers of people with severe mental illness	Mental health illnesses (various)	Qualitative
Pattanayak, R. D., Jena, R., Tripathi, M. and Khandelwal, S. K. (2010). Assessment of burden in caregivers of Alzheimer’s disease from India. *Asian Journal of Psychiatry*, **3**, 112–116.	32 patient–caregiver dyads India	Alzheimer’s disease	Quantitative
Gresswell, I., Lally, L., Adamis, D. and McCarthy, G. M. (2018). Widening the net: exploring social determinants of burden of informal carers. *Irish Journal of Psychological Medicine*, **35**, 43–51.	53 Irish family carers, unspecified	Dementia or other chronic illness	Quantitative
McAuliffe, L., Ong, B. and Kinsella, G. (2018). Mediators of burden and depression in dementia family caregivers: kinship differences. *Dementia*.	134 family caregivers	Dementia	Quantitative
Stadtmann, M. P., Maercker, A., Binder, J. and Schnepp, W. (2018). Mastering life together-symptom management, views, and experiences of relatives of persons with CPTSD: a grounded theory study. *Journal of Patient-Reported Outcomes*, **2**, 1–13.	17 self-declared ‘relatives’	Complex posttraumatic stress disorder	Qualitative
Viñas‐Diez, V., Turró‐Garriga, O., Portellano‐Ortiz, C., Gascón‐Bayarri, J., Reñé‐Ramírez, R., Garre‐Olmo, J. *et al.* (2017). Kinship and cohabitation in relation to caregiver burden in the context of Alzheimer's disease: a 24‐month longitudinal study. *International Journal of Geriatric Psychiatry*, **32**, e72–e82.	275 Alzheimer’s disease family primary caregivers	Alzheimer	Quantitative
Chow, E. O. W. and Ho, H. C. (2015). Caregiver strain, age, and psychological well-being of older spousal caregivers in Hong Kong. *Journal of Social Work*, **15**, 479–497.	112 spousal caregivers aged 55 and over in Hong Kong	Not specified	Quantitative
Tuomola, J., Soon, J., Fisher, P. and Yap, P. (2016). Lived experience of caregivers of persons with dementia and the impact on their sense of self: a qualitative study in Singapore. *Journal of Cross-Cultural Gerontology*, **31**, 157–172.	6 Chinese female spousal caregivers	Dementia	Qualitative
Mausbach, B. T., Chattillion, E. A., Ho, J., Flynn, L. M., Tiznado, D., von Känel, R. *et al.* (2014). Why does placement of persons with Alzheimer’s disease into long-term care improve caregivers’ well-being? Examination of psychological mediators. *Psychology and Aging*, **29**(4), 776.	126 spousal Alzheimer’s disease family caregivers	Alzheimer	Quantitative
Williams, K. L., Morrison, V. and Robinson, C. A. (2014). Exploring caregiving experiences: caregiver coping and making sense of illness. *Aging & Mental Health*, **18**, 600–609.	13 family caregivers	Stroke and dementia	Qualitative
Abu Bakar, S. H., Weatherley, R., Omar, N., Abdullah, F. and Mohamad Aun, N. S. (2014). Projecting social support needs of informal caregivers in Malaysia. *Health & Social Care in the Community*, **22**, 144–154.	175 family caregivers	Unspecified (chronically ill/disability)	Quantitative
Orpin, P., Stirling, C., Hetherington, S. and Robinson, A. (2014). Rural dementia carers: formal and informal sources of support. *Ageing & Society*, **34**(2), 185–208.	18 rural primary caregivers (unspecified)	Dementia	Qualitative
Gibbons, C., Creese, J., Tran, M., Brazil, K., Chambers, L., Weaver, B. and Bédard, M. (2014). The psychological and health consequences of caring for a spouse with dementia: a critical comparison of husbands and wives. *Journal of Women & Aging*, **26**, 3–21.	65 spouses (husbands/wives) caring for someone with Alzheimer’s disease	Dementia	quantitative
Daley, R. T., Sugarman, M. A., Shirk, S. D. and O’Connor, M. K. (2018). Spared emotional perception in patients with Alzheimer’s disease is associated with negative caregiver outcomes. *Aging & Mental Health*, **22**(5), 595–602.	28 spousal caregivers and 30 controls	Alzheimer’s	Quantitative
van Groenou, M. I. B., de Boer, A. and Iedema, J. (2013). Positive and negative evaluation of caregiving among three different types of informal care relationships. *European Journal of Ageing*, **10**, 301–311.	1685 Dutch family caregivers	Multiple	Quantitative
Alpass, F., Pond, R., Stephens, C., Stevenson, B., Keeling, S. and Towers, A. (2013). The influence of ethnicity and gender on caregiver health in older New Zealanders. *Journals of Gerontology Series B: Psychological Sciences and Social Sciences*, **68**, 783–793.	2155 New Zealand family carers	Unspecified	Quantitative
Slachevsky, A., Budinich, M., Miranda-Castillo, C., Núñez-Huasaf, J., Silva, J. R., Muñoz-Neira, C. *et al.* (2013). The CUIDEME Study: determinants of burden in Chilean primary caregivers of patients with dementia. *Journal of Alzheimer’s Disease*, **35**(2), 297–306.	292 Chilean family caregivers	Dementia	Quantitative
Pöysti, M. M., Laakkonen, M. L., Strandberg, T., Savikko, N., Tilvis, R. S., Eloniemi-Sulkava, U. and Pitkälä, K. H. (2012). Gender differences in dementia spousal caregiving. *International Journal of Alzheimer’s Disease*, 2012, 162960.	335 dyads of wife– husband caregivers	Dementia	Quantitative
Quinn, C., Clare, L. and Woods, R. T. (2012). What predicts whether caregivers of people with dementia find meaning in their role? *International Journal of Geriatric Psychiatry*, **27**(11), 1195–1202.	447 informal caregivers	Dementia	Quantitative
Lai, D. W. (2012). Effect of financial costs on caregiving burden of family caregivers of older adults. *Sage Open*, **2**(4), 2158244012470467.	448 informal caregivers	Unspecified	Quantitative
Tsai, C.-F., Hwang, W.-S., Lee, J.-J., Wang, W.-F., Huang, L.-C., Huang, L.-K. *et al.* (2021) Predictors of caregiver burden in aged caregivers of demented older patients. *BMC Geriatrics*, **21**, 1–9.	328 informal caregiver–patient dyads.	Dementia	Quantitative
Papastavrou, E., Charalambous, A., Tsangari, H. and Karayiannis, G. (2012). The burdensome and depressive experience of caring: what cancer, schizophrenia, and Alzheimer’s disease caregivers have in common. *Cancer Nursing*, **35**, 187–194.	410 family caregivers	Cancer, Schizophrenia, and Alzheimer	Quantitative
Clare, L., Nelis, S. M., Whitaker, C. J., Martyr, A., Markova, I. S., Roth, I. *et al.* (2012). Marital relationship quality in early-stage dementia: perspectives from people with dementia and their spouses. *Alzheimer Disease & Associated Disorders*, **26**, 148–158.	54 spousal caregivers and 54 control couples	Dementia	Quantitative
Shanley, C., Russell, C., Middleton, H. and Simpson-Young, V. (2011). Living through end-stage dementia: the experiences and expressed needs of family carers. *Dementia*, **10**, 325–340.	15 informal carers	End-stage dementia	Quantitative
Papastavrou, E., Tsangari, H., Karayiannis, G., Papacostas, S., Efstathiou, G. and Sourtzi, P. (2011). Caring and coping: the dementia caregivers. *Aging & Mental Health*, **15**, 702–711.	172 Greek family caregivers	Dementia	Quantitative
Innes, A., Abela, S. and Scerri, C. (2011). The organisation of dementia care by families in Malta: the experiences of family caregivers. *Dementia*, **10**, 165–184.	17 family caregivers from Malta	Dementia	Qualitative
Nordtug, B. and Holen, A. (2011). Similarities and differences in caring burden of home dwellers with partners suffering from chronic obstructive pulmonary disease or dementia. *Home Health Care Management & Practice*, **23**, 93–101.	206 Norwegian spousal carers	Chronic obstructive pulmonary disease (COPD) or dementia	Quantitative
Lai, D. W. and Thomson, C. (2011). The impact of perceived adequacy of social support on caregiving burden of family caregivers. *Families in Society*, **92**, 99–106.	340 Canadian family caregivers aged above 65	Unspecified	Quantitative
Stanley, S., Balakrishnan, S. and Ilangovan, S. (2017). Psychological distress, perceived burden and quality of life in caregivers of persons with schizophrenia. *Journal of Mental Health*, **26**, 134–141.	75 primary family caregivers of persons with schizophrenia in India	Schizophrenia	Quantitative cross-sectional design and survey
Baker, K. L., Robertson, N. and Connelly, D. (2010). Men caring for wives or partners with dementia: masculinity, strain and gain. *Aging & Mental Health*, **14**, 319–327.	70 male caregivers	Dementia	Quantitative
Papastavrou, E., Tsangari, H., Kalokerinou, A., Papacostas, S. S. and Sourtzi, P. (2009). Gender issues in caring for demented relatives. *Health Science Journal*, **3**, 41–53.	172 Cyprus family primary caregivers	Dementia	Quantitative
Arango Lasprilla, J. C., Moreno, A., Rogers, H. and Francis, K. (2009). The effect of dementia patient’s physical, cognitive, and emotional/behavioral problems on caregiver well-being: findings from a Spanish-speaking sample from Colombia, South America. *American Journal of Alzheimer's Disease & Other Dementias*, **24**, 384–395.	73 Colombian family caregivers	Dementia	Quantitative
Sun, F., Lee Roff, L., Klemmack, D. and Burgio, L. D. (2008). The influences of gender and religiousness on Alzheimer disease caregivers’ use of informal support and formal services. *Journal of Aging and Health*, **20**, 937–953.	720 family caregivers	Alzheimer	Quantitative
Quinn, C., Clare, L., Pearce, A. and Van Dijkhuizen, M. (2008). The experience of providing care in the early stages of dementia: an interpretative phenomenological analysis. *Aging and Mental Health*, **12**, 769–778.	34 spouses or partners of people with a diagnosis of early-stage dementia	Early stages of dementia	Qualitative
Brown, J. and Chen, S. L. (2008). Help-seeking patterns of older spousal caregivers of older adults with dementia. *Issues in Mental Health Nursing*, **29**, 839–852.	20 spousal caregivers	Dementia	Qualitative
Dahlberg, L., Demack, S., and Bambra, C. (2007). Age and gender of informal carers: a population‐based study in the UK. *Health & Social Care in the Community*, **15**, 439–445.	a 3% random sample of the 2001 UK Census. The sample size was 1 825 595. Of this sample, 10% were reported to be carers.	Unspecified	Quantitative
Ducharme, F., Lévesque, L., Zarit, S. H., Lachance, L. and Giroux, F. (2007). Changes in health outcomes among older husband caregivers: a one-year longitudinal study. *The International Journal of Aging and Human Development*, **65**, 73–96.	232 older husband caregivers	Unspecified	Quantitative, longitudinal
Calasanti, T. and Bowen, M. E. (2006). Spousal caregiving and crossing gender boundaries: maintaining gendered identities. *Journal of Aging Studies*, **20**, 253–263.	22 primary spousal caregivers	Alzheimer	Qualitative
Adams, K. B., Smyth, K. A. and McClendon, M. J. (2005). Psychosocial resources as moderators of the impact of spousal dementia caregiving on depression. *Journal of Applied Gerontology*, **24**, 475–489.	441 caregivers and 251 Non-caregivers	Alzheimer	Quantitative
Malhotra, C., Malhotra, R., Østbye, T., Matchar, D. and Chan, A. (2012). Depressive symptoms among informal caregivers of older adults: insights from the Singapore Survey on Informal Caregiving. *International Psychogeriatrics*, **24**, 1335–1346.	1190 dyads caregivers and receivers	Multiple	Quantitative
Gaugler, J. E., Anderson, K. A., Leach, C. R., Smith, C. D., Schmitt, F. A. and Mendiondo, M. (2004). The emotional ramifications of unmet need in dementia caregiving. *American Journal of Alzheimer's Disease & Other Dementias*, **19**, 369–380.	694 informal caregivers	Dementia	Quantitative
Beeson, R. A. (2003). Loneliness and depression in spousal caregivers of those with Alzheimer’s disease versus non-caregiving spouses. *Archives of Psychiatric Nursing*, **17**, 135–143.	49 Alzheimer spousal carers (AD) and 52 spousal non-caregivers	Alzheimer	Quantitative
Perry, J. (2002). Wives giving care to husbands with Alzheimer’s disease: a process of interpretive caring. *Research in Nursing & Health*, **25**, 307–316.	20 spousal carers (wives)	Alzheimer	Qualitative
Chow, E. O. W. and Ho, H. C. (2015). Caregiver strain, age, and psychological well-being of older spousal caregivers in Hong Kong. *Journal of Social Work*, **15**, 479–497.	112 spousal caregivers from Hong Kong	Not specified	Quantitative
Gonçalves-Pereira, M., Carmo, I., da Silva, J. A., Papoila, A. L., Mateos, R. and Zarit, S. H. (2010). Caregiving experiences and knowledge about dementia in Portuguese clinical outpatient settings. *International Psychogeriatrics*, **22**, 270–280.	99 primary caregivers	Dementia	Quantitative
Gresswell, I., Lally, L., Adamis, D. and McCarthy, G. M. (2018). Widening the net: exploring social determinants of burden of informal carers. *Irish Journal of Psychological Medicine*, **35**, 43–51.	53 carers Ireland	Not specified	Quantitative
Shikimoto, R., Sado, M., Ninomiya, A., Yoshimura, K., Ikeda, B., Baba, T. *et al.* (2018). Predictive factors associated with psychological distress of caregivers of people with dementia in Japan: a cross-sectional study. *International Psychogeriatrics*, **30**, 1089–1098.	Japan, 1437 people with dementia-caregiver dyads	Dementia	Quantitative
Temple, J. B. and Dow, B. (2018). The unmet support needs of carers of older Australians: prevalence and mental health. *International Psychogeriatrics*, **30**, 1849–1860.	25 555 primary carers Australia	Multiple	Quantitative
Collins, C. and Jones, R. (1997). Emotional distress and morbidity in dementia carers: a matched comparison of husbands and wives. *International Journal of Geriatric Psychiatry*, **12**, 1168–1173.	48 UK spousal carers	Dementia	Quantitative
Luderowski, A. and Boden, Z. V. (2020). Love and incomprehensibility: the hermeneutic labour of caring for and understanding a loved one with psychosis. *Health*, **24**, 737–754.	10 UK informal carer for someone experiencing psychosis	Psychosis	Qualitative
Wayland, S., Coker, S. and Maple, M. (2021). The human approach to supportive interventions: the lived experience of people who care for others who suicide attempt. *International Journal of Mental Health Nursing*, **30**, 667–682.	758 family carers of someone who has attempted suicide	Suicide	Mixed
Vasileiou, K., Barnett, J., Barreto, M., Vines, J., Atkinson, M., Lawson, S. *et al.* (2017) Experiences of loneliness associated with being an informal caregiver: a qualitative investigation. *Frontiers in Psychology*, **8**, 585.	16 family caregivers	Mixed (dementia, frailty due to old age, multiple sclerosis, depression, autism)	Qualitative
Östman, M., Wallsten, T. and Kjellin, L. (2005). Family burden and relatives' participation in psychiatric care: are the patient’s diagnosis and the relation to the patient of importance? *International Journal of Social Psychiatry*, **51**, 291–301.	455 close relatives caregivers	Psychoses, affective disorders and ‘other diagnoses’	Quantitative
McGaw, V. E., Reupert, A. E. and Maybery, D. (2020). Partners of veterans with PTSD: parenting and family experiences. *Families in Society*, **101**, 456–468.	8 female partner caregivers of Australian veterans	PTSD	Qualitative
Roth, D. L., Dilworth-Anderson, P., Huang, J., Gross, A. L. and Gitlin, L. N. (2015). Positive aspects of family caregiving for dementia: differential item functioning by race. *Journals of Gerontology Series B: Psychological Sciences and Social Sciences*, **70**, 813–819.	642 family caregivers	Dementia	Quantitative
Brown, S. L., Smith, D. M., Schulz, R., Kabeto, M. U., Ubel, P. A., Poulin, M., *et al.* (2009). Caregiving behavior is associated with decreased mortality risk. *Psychological Science*, **20**, 488–494.	3376 family caregivers	Multiple	Quantitative
O’Reilly, D., Connolly, S., Rosato, M. and Patterson, C. (2008). Is caring associated with an increased risk of mortality? A longitudinal study. *Social Science & Medicine*, **67**, 1282–1290.	Health of caregivers recorded in the 2001 Northern Ireland Census	Multiple	Quantitative
Roth, D. L., Perkins, M., Wadley, V. G., Temple, E. M. and Haley, W. E. (2009). Family caregiving and emotional strain: associations with quality of life in a large national sample of middle-aged and older adults. *Quality of Life Research*, **18**, 679–688.	43 099 family caregivers	Multiple	Quantitative
Dilworth-Anderson, P., Brummett, B. H., Goodwin, P., Williams, S. W., Williams, R. B. and Siegler, I. C. (2005). Effect of race on cultural justifications for caregiving. *The Journals of Gerontology Series B: Psychological Sciences and Social Sciences*, **60**, S257–S262.	48 African American and 121 White caregivers.	Multiple	Quantitative
McCallum, T. J., Sorocco, K. H. and Fritsch, T. (2006). Mental health and diurnal salivary cortisol patterns among African American and European American female dementia family caregivers. *The American Journal of Geriatric Psychiatry*, **14**, 684–693.	30 African American and 24 European American female dementia family caregivers and 48 African American and 15 European American non-caregivers	Dementia	Quantitative

As outlined in Table 3, the review identified three main themes: (i) Negative Implications of Caring for Family Members with Mental Health Diseases, (ii) Positive Implications of Caring for Family Members with Mental Health Diseases and (iii) Moderating Factors that can Contribute to the Family Carer’s Wellbeing. We now discuss these three themes in more detail.

**Table 3:** Summary of themes and key results

Theme: name	Theme: description	Theme: prevalence	Example: article
Negative Implications of Caring for Family Members with Mental Health Diseases	Studies that provide qualitative or quantitative evidence for adverse physical and/or psychological consequences of family caregiving are included in this theme. Examples for adverse physical and/or psychological consequences are e.g. depression, burden of care, muscular dystrophy, disc prolapses, increased mortality rates, etc.	54.24% of articles described adverse physical and/or psychological consequences of family caregiving	Viñas‐Diez, V., Turró‐Garriga, O., Portellano‐Ortiz, C., Gascón‐Bayarri, J., Reñé‐Ramírez, R., Garre‐Olmo, J., *et al.* (2017). Kinship and cohabitation in relation to caregiver burden in the context of Alzheimer’s disease: a 24‐month longitudinal study. *International Journal of Geriatric Psychiatry*, **32**, e72–e82.
Positive Implications of Caring for Family Members with Mental Health Diseases	Studies that provide qualitative or quantitative evidence for beneficial physical and/or psychological consequences of family caregiving are included in this theme. Examples for beneficial physical and/or psychological consequences are e.g. personal growth, maturity, increased resilience, reduced all-cause mortality rates, etc.	17.39% of articles investigated positive and beneficial effects of family caregiving.	Quinn, C., Clare, L. and Woods, R. T. (2012). What predicts whether caregivers of people with dementia find meaning in their role? *International Journal of Geriatric Psychiatry*, **27**, 1195–1202.
Moderating Factors that can Contribute to the Family Carer’s Wellbeing	Studies that examine factors that contribute to family caregivers’ wellbeing and family caregivers’ needs are included in this theme. Examples for unmet needs are physical, social and financial support.	A total of 47.82% examined how moderating factors contribute to family caregivers’ wellbeing.	Temple, J. B. and Dow, B. (2018). The unmet support needs of carers of older Australians: prevalence and mental health. *International Psychogeriatrics*, **30**, 1849–1860.

### Negative implications of caring for family members with mental health diseases

A major theme was the negative impact on the family caregiver’s physical and mental health. Most of the articles included in this review examined clinical levels of psychological distress in family carers (33.7%; 31 studies) and depression and depressive symptoms in relation to family caregiving (21.17%, 20 studies). This indicates that providing primary care for family members can have problematic consequences on the carers’ wellbeing. For example, Mausbach *et al.*(Mausbach *et al.*, 2014) conducted a longitudinal study comparing the individual experiences of 126 spousal carers for Alzheimer patients. The placement of the care receiver into care homes was associated with significant reductions in depressive symptoms, activity restriction and an increase in personal mastery in caregivers. These results are supported by studies that examined levels of psychological distress and depressive symptoms in primary and secondary caregivers across different mental health diseases and different cultures [i.e. (Colvez et al., 2002; Gonçalves-Pereira *et al.*, 2020; Zwar *et al.*, 2020)].

However, while primary care for family members can have negative consequences on carer wellbeing, sociodemographic factors can also determine the extent to which individuals may be affected by such negative consequences. For example, 32 (34.78%) of the reviewed studies indicated that gender significantly impacts on the care givers self-perceived burden of care. The results from these studies are consistent in suggesting that female family caregivers experience higher levels of psychological distress, shame and caregiver burden [i.e. (Pattanayak *et al.*, 2010; Chiang *et al.*, 2015; Gresswell *et al.*, 2018; Avdikou *et al.*, 2019; Brites *et al.*, 2020)]. This trend was evident across different cultures and applied to various mental health disorders equally (Papastavrou *et al.*, 2009; Nordtug and Holen, 2011; Pöysti *et al.*, 2012). In addition, if wives cared for their husbands, they experienced lower marital satisfaction, while the opposite effect was observed in husbands caring for their wives (Beeson, 2003; Dahlberg *et al.*, 2007; Lai, 2012; Choi, 2018). The literature around this phenomenon suggests that women feel more responsible for providing care and as a result are left with multiple competing roles (Calasanti and Bowen, 2006; Dahlberg *et al.*, 2007). Women are therefore more likely to experience a loss of self and loneliness when caring for a family member making them more susceptible to caregiver depression, whereas men are more comfortable with letting others assume the carer role, and are more self-reliant on themselves and others to provide social support (Calasanti and Bowen, 2006; Brown and Chen, 2008; Sun *et al.*, 2008; Ruisoto *et al.*, 2020).

The role of the carer and their relationship with the care-receiver was also found to impact on individual wellbeing. Specifically, research suggests that close ties such as being a spouse and/or living together with a mentally ill person in an acute setting determines the caregiver’s experiences of burden of care. For example, in their quantitative study with 455 close relatives, found that burden was particularly high among spouses, as they feel more isolated and have less leisure time than other family carers. In addition, findings suggest that spouses who care for partners with mental health disorders are exposed to additional stressors that originate from an internal conflict of roles were they are perceived to ‘hold it all together’ in their relationship [(Casarez *et al.*, 2019), p. 393]. In this sense, spouses need to handle challenging behaviours such as reckless spending or rageful and hypercritical behaviour, minimizing any issues that might arise during social encounters (Casarez *et al.*, 2019). Besides the emotional burden, spousal carers may also experience financial difficulties as they often give up their work to care for their loved ones. This poses additional problems as financial uncertainty contributes to clinically significant levels of distress, caregiver burden and depression among family carers (Lai, 2012; Abu Bakar *et al.*, 2014; Temple and Dow, 2018; Wayland *et al.*, 2021). Therefore, spouses may be at risk of compromising their own wellbeing when delivering primary care, due to greater emotional and financial strain.

Another key determinant in the prediction of family carer’s wellbeing is age. Studies in this review suggest that challenging experiences in relation to family caring were perceived to be more difficult or worse for older carers (Chiang *et al.*, 2015; Chow and Ho, 2015: Tsai *et al.*, 2021). Essentially, elderly family carers may not understand mental health issues or have the stamina required to overcome the challenges of caring, for example accessing formal support (Chiang *et al.*, 2015; Greenwood *et al.*, 2019). In addition, their own health conditions can compromise the ability to perform physical tasks, and thus diminish their social circle. Potential sources of social support can also decrease with age, while their own health conditions can make it harder to leave their home, and engage in leisure activities or make new friends.

### Positive implications of caring for family members with mental health diseases

While most literature examines the negative psychological and physical consequences of caregiving on the carer, there is an increasing emphasis on the positives that bring balance to this point of view. A total of 16 studies (17.39%) in this review investigated the full range of caregiving experiences, including potential positive aspects and benefits of caregiving. Essentially, caregiving can be a rewarding experience that facilitates personal growth, maturity and resilience (Netto *et al.*, 2009; Sánchez-Izquierdo *et al.*, 2015; Quinn *et al.*, 2015) with population-based surveys indicating that caregiving may relate to significantly reduced all-cause mortality rates in comparison to a matched sample of non-caregivers (Brown *et al.*, 2009; O’Reilly *et al.*, 2008). Caregivers often report feeling gratitude and a sense of mastery, meaningfulness and coherence in their existence (Cohen *et al.*, 2002; Quinn *et al.*, 2012; Cheng *et al.*, 2017). Therefore, the findings that suggest positive aspects of caregiving to be inversely related to burden and depression are unsurprising [i.e. (Hilgeman *et al.*, 2007)].

Theoretical models offer different explanations to understand these positive changes in the caregiver’s life. For example, the adaption stress and coping model (Folkman, 1997), suggests that positive psychological states are associated with finding positive meaning in adversity, and helping the individual to make sense of the situation. This can result in the caregiver identifying positive changes in their life (Park, 2010). Fredrickson *et al.*(Fredrickson *et al.*, 2003) take a similar approach in their broaden-and-build theory by suggesting that positive emotions contribute to cognitive broadening, which then widens the individual’s attention, thinking and behaviour. This broadening effect helps foster a range of adaptive and durable personal resources that contribute to the development of resilience (Fredrickson, 2004). Similarly, theories that are based on benefit-finding imply that adaptive coping emerges over time as a way of responding to stressful circumstances. This enables personal growth and fosters resilience.

In addition to situational and personal factors, literature indicates that adaptive coping also depends upon demographic characteristics. For example, caregiver personality characteristics such as extraversion and agreeableness along with social support (especially support from loved ones) are associated with positive aspects of caregiving (Koerner *et al.*, 2009). Ethnicity can also impact on the experience of positive aspects in caregiving. Studies show that African Americans report more positive caregiving outcomes associated with active coping styles and greater resilience in comparison to Caucasians (Dilworth-Anderson *et al.*, 2005; McCallum *et al.*, 2006; Merritt *et al.*, 2011). This is consistent with studies in which African American family caregivers were less affected by depression, caregiver burden and strain than white family caregivers (Haley *et al.*, 2004). A study by Roth *et al.* (2015) also found that African American family carers felt more capable at appreciating and developing a positive attitude towards life when compared to Caucasians. This positive attitude towards care is explained as a reflection of long-standing traditions of the African American community. This attitude is cultivated in early life through racial socialization and maintained in adulthood life through spiritual and cultural beliefs, especially when facing adversities or overcoming hardships (McLoyd *et al.*, 2005). Roff *et al.* (2004) outlined similar findings but added that religiosity partially mediates the relationship between ethnicity and the elevated positive attitudes in caregiving among African Americans. Although symptoms of depression were not associated with baseline levels of positive aspects in caregiving, Roff *et al.*(Roff *et al.*, 2004) found that lower anxiety levels, less worrying and lower socioeconomic status among African American caregivers contributed to higher levels of positive aspects in caregiving. In conclusion, positive aspects of caregiving may be related to coping and having a more optimistic outlook on life.

### Moderating factors that can contribute to the family carers’ wellbeing

A total of 44 studies (47.82%) suggest that tailored means of support are pivotal in improving family carers’ physical and mental wellbeing. Here, a particular focus is placed on examining the individual needs of the family caregiver and how these may be addressed (Temple and Dow, 2018). Findings from the review suggest that the most prevalent types of unmet needs include financial, physical and social support [i.e. (Highet *et al.*, 2005; Temple and Dow, 2018; Brickell *et al.*, 2019; Casarez *et al.*, 2019; Jones *et al.*, 2019; Alasmee and Hasan, 2020; Anchan and Janardhana, 2020; Brites *et al.*, 2020; Lai *et al.*, 2020; Teles *et al.*, 2021)]. Temple and Dow’s (2018) findings indicate that having any unmet needs for support increased the likelihood of the carer experiencing psychological distress two-fold. With each successive unmet need for support, the odds of psychological distress increased another 1.37 times. Therefore, besides the previously discussed negative effect of financial burden (7 studies; 7.61%), maintaining social connections (17 studies; 18.48%) and receiving professional or social support (16 studies; 17.39%), unmet needs are key factors in understanding and addressing the carer’s physical and psychological wellbeing.

Increasing social resources was found to reduce the risk of suffering from poor mental health in family caregivers for patients with different mental illnesses (Gaugler *et al.*, 2004; Griffin *et al.*, 2017; Otero *et al.*, 2019). This may be explained by feelings of social connectedness counteracting the effects of loneliness and social isolation that are associated with depression and depressive symptoms in family carers (Beeson, 2003; Vasileiou *et al.*, 2017; McAuliffe *et al.*, 2018). In this sense, empathy and social connections with family and friends as well as healthcare staff can provide a source of strength and a sense of mastery in one’s predicament (Shanley *et al.*, 2011). Peer support groups may be particularly relevant in reducing the burden of care by providing the family carer with a feeling of social connectedness with individuals who share similar experiences (Shanley *et al.*, 2011). In addition, peer support groups give family carers the space and opportunity to reflect on their own caring routine, helping them cope more effectively with the competing demands of care (Stanley *et al.*, 2017).

In addition to peer support groups, research suggests that smartphone technologies (i.e. applications) can help improve and maintain family carers’ physical and mental health. For example, Casarez *et al.*(Casarez *et al.*, 2019) report that using mental health (mHealth) apps leads to a reduction of carer burden and distress and an increase in social contacts. They not only improve the relationship between carer and care-receiver but help them to manage the administration of medication and contribute to mental health literacy. Specifically, mHealth apps can provide information for family carers on how to manage symptoms of mental health disorders, what to do in difficult situations, provide constructive practices and alert users to the need for professional interventions. This is important because information about mental health symptoms can foster an understanding of the care receivers’ experiences and reduce burden and distress in caregivers [i.e. (Vasileiou *et al.*, 2017; Brickell *et al.*, 2019; Casarez *et al.*, 2019; Jones *et al.*, 2019; Alasmee and Hasan, 2020; Anchan and Janardhana, 2020; Hahn *et al.*, 2020; Lai *et al.*, 2020; McGaw *et al.*, 2020; Wayland *et al.*, 2021)]. However, while mental health literacy is crucial in improving caregiver wellbeing, studies have found that in certain situations, carers are not interested in information about the mental health illness of the care receiver (Williams *et al.*, 2014). Rather, Williams *et al.*(Williams *et al.*, 2014) suggests that caregivers adopt active information-seeking techniques to not only deal with current problems and to increase their sense of control, but avoid considering future logistics of caregiving when they feel helpless or overwhelmed with stress. Thus, caregiving stressors affect the extent to which caregivers may adequately utilize the information and service provisions available. In conclusion, for them to be effective, resources must be tailored to the caregivers’ individual needs.

Importantly, cultural differences need to be taken into consideration when tailoring support [i.e. (Sun *et al.*, 2008; Chow and Ho, 2015; Meyer *et al.*, 2015; Tuomola *et al.*, 2016; Mahomed and Pretorius, 2020; Kabitsi and Powers, 2002)]. This review suggests that cultural differences impact on patterns of help-seeking behaviour, access to sources of formal and informal support, the search for information about the mental illness and the motivation to provide family care (Kabitsi and Powers, 2002; Mahomed and Pretorius, 2020). For example, Mahomed and Pretorius (Mahomed and Pretorius, 2020) found that male South Africans in low-income communities utilize a collective approach towards caregiving, relying on wider informal social networks instead of institutional and formal care services. Similar results were evident in studies that compared motives for family caregiving in Greece and America (Kabitsi and Powers, 2002). This study found that Greek and American carers were motivated by different factors. Greek families understood themselves in a more relational and largely family-interwoven frame, while American family carers were more motivated by financial reasons to provide family care. It can therefore be concluded that cultural differences in caregiving motivations may result in adaptive or maladaptive outcomes for the caregiver and care receiver. Here, studies suggest that cultural and religious values often correlate and overlap with one another. Several studies outlined the importance of religion and spirituality in helping family caregivers to manage the stress of caregiving [i.e. (Meyer *et al.*, 2015; Robinson-Lane *et al.*, 2021)]. For example, Tuomola *et al.* (Tuomola *et al.*, 2016), Chan (Chan, 2010) and Hinton *et al.* (Hinton *et al.*, 2008) outlined how Confucian principles that highlight the importance of closeness between family members are reflected in family carers’ reasoning processes. By drawing on the collectivistic cultures of East Asia that espouse a set of values and beliefs which promote maintenance of harmony with individuals and with the environment, family carers could overcome obstacles and emotional difficulties. Similar results are supported in Meyer *et al.*’s (Meyer *et al.*, 2015) examination of Christian family carers who relied on their faith to cope with challenging situations. Community support and doctrinal beliefs about God’s will helped caregivers to cope with distress and burden. In conclusion, the studies presented in this review suggest that it may be valuable to include an understanding of cultural values and an individual’s spirituality and religion as components of tailored means of support for family caregivers.

## DISCUSSION AND CONCLUSION

This scoping review was conducted to understand the effects of caring for family members with mental health illnesses on caregivers’ wellbeing. The findings suggest that mental and physical wellbeing of family caregivers may depend upon a combination of situational and sociodemographic characteristics.

The review identified elderly, female and spousal primary-caregivers as a group who are at risk of experiencing mental and physical health. Primary caregivers, in comparison to secondary caregivers, suffer higher levels of distress, have less leisure time and smaller social circles. In addition, the findings indicate that there are gender differences that also need to be taken into consideration. The scoping review found that female spousal carers may view caring for their partner as their duty, try to be less dependent on support from others and take on multiple roles to fill the gaps that the mental illness of their partner has caused in comparison to their male counterparts, In doing so, female spousal carers may experience higher levels of distress and caregiver burden than male family caregivers. Spousal family caregivers often give up their work. This is linked to financial uncertainty and elevated levels of distress and depressive symptoms. In this sense, it is possible that some factors associated with caring for family members such as limited temporal resources may contribute to and trigger further stressors such as financial uncertainty. Similarly, elderly family caregivers tend to experience lower levels of wellbeing as they often do not have the stamina required for caring responsibilities, and experience diminishing social circles besides suffering from their own health issues. Therefore, the results suggest that for older caregivers, there are a range of underlying stressors that decrease the individual’s overall quality of life and life satisfaction while increasing the burden of care.

Nevertheless, caregiving can also be a rewarding experience that fosters meaning, resilience and personal growth in the caregiver. Therefore, the present results suggest that the negative effects of caregiving can be balanced by the individuals’ personality, social support, religiosity and culture. Specifically, high levels of agreeableness and extraversion in combination with social support relate to lower levels of caregiver stress and burden. Social resources were found to play a particularly important role because support from peers counteracts the loneliness and isolation that can lead to depression. In this sense, social resources combined with personality traits and social skills may be important in maintaining life satisfaction and quality of life. Also, positive attitudes towards life coupled with religious or spiritual beliefs helped improve the family caregivers’ wellbeing because spirituality and religiosity provide a framework through which caregiving experiences can be interpreted in meaningful ways. This supports the idea that the meaningful interpretation of one’s circumstances is an important skill that can help give family caregivers a positive outlook on their life and understand difficult circumstances through multiple perspectives.

However, despite the findings from this review, several limitations need to be taken into consideration. The present review did not examine demands, capabilities and meaning at the community level, nor did it consider the care receiver’s adaptation and adjustment to their mental illness. The scoping review did not seek to assess the studies included regarding their quality or effect sizes. We also acknowledge that the sample of articles is skewed towards the challenges and demands of family caregivers for dementia (38 articles, 41.3%) and Alzheimer patients (13 articles, 14.13%). While the scholarship reviewed suggests that there is little difference in caregiver experience between conditions, it may still be possible that other unique aspects of the caregiver experiences in relation to other mental health illnesses have not been covered. In this sense, the present review included caregivers of individuals with different pathologies but did not necessarily differentiate between their experiences. Future research may therefore wish to focus on the experiences of family caregivers looking after an individual with specific mental health conditions such as depression or schizophrenia. In addition, as the care receiver’s health can also influence the caregiver’s quality of life, only few studies in the review discussed the caregivers’ overall quality of life. This is problematic as it remains unclear whether the caregiver has lower self-rated quality of life besides scoring high on e.g. caregiver burden, and caregiver distress. This limitation can be addressed in future reviews focussing on examining and comparing specific measures such as self-rated quality of life. In addition, the scope of future reviews could include research that examines interventions intending to improve family caregivers’ wellbeing.

Despite these limitations, the present review achieved its research objective by providing an overview of how family caregiving affects the caregivers physical, mental and social wellbeing, and the best ways to support family carers. While caregiving can have a negative impact on the physical and mental wellbeing of the carer, specific sociodemographic, personal and situational factors can work as a buffer. Therefore, support for family caregivers must be tailored to individual needs, taking into consideration personality, particular circumstances, along with cultural and personal beliefs.
